# Using Evolutionary Analyses to Refine Whole-Genome Sequence Match Criteria

**DOI:** 10.3389/fmicb.2022.797997

**Published:** 2022-06-16

**Authors:** Arthur W. Pightling, Hugh Rand, James Pettengill

**Affiliations:** Biostatistics and Bioinformatics Staff, Office of Analytics and Outreach, Center for Food Safety and Applied Nutrition, U.S. Food and Drug Administration, College Park, MD, United States

**Keywords:** whole-genome sequence, evolutionary analyses, *Salmonella enterica*, outbreak investigation, evolutionary rate, resident strain, match, closely related genetically

## Abstract

Whole-genome sequence databases continue to grow. Collection times between samples are also growing, providing both a challenge for comparing recently collected sequence data to historical samples and an opportunity for evolutionary analyses that can be used to refine match criteria. We measured evolutionary rates for 22 *Salmonella enterica* serotypes. Based upon these measurements, we propose using an evolutionary rate of 1.97 single-nucleotide polymorphisms (SNPs) per year when determining whether genome sequences match.

## Background

Whole-genome sequence (WGS) databases exist for many important bacterial pathogens^1^ and the analysis of such data is routine during surveillance and outbreak investigations (Allard et al., 2016). WGS data is commonly used to identify matching isolates. (i.e., isolates that arose from a recent source of contamination; Pightling et al., 2018). Determination of isolate matches usually relies on estimates of genomic distances and tree topologies; generally, these determinations do not incorporate collection times of isolates (Pightling et al., 2018; Wang et al., 2018). Omitting this temporal information is satisfactory when the time spans between collection dates are small, but evolutionary changes could lead to incorrectly inferring mismatches when the time spans are large. That is, isolates collected only a year apart are expected to have fewer genomic differences than isolates that were collected 10 years apart; applying the same genomic distance match criteria to each pair may not be appropriate. Thus, it is necessary to develop match criteria that incorporate the time spans separating collection times. Here, we present the estimated evolutionary rates of bacteria representing 22 *Salmonella enterica* serotypes that were collected from US food manufacturers and show how those rates can be incorporated into distance analyses that are used to assess matches between *S. enterica* genomes.

## Materials and Methods

### Dataset

We identified 15,580 *S. enterica* genome sequences that: (1) originated in the United States from 2014 to 2019, (2) were generated on Illumina platforms, and (3) were submitted to the National Center for Biotechnology Information’s Pathogen Detection (NCBI’s) portal^2^ by the U.S. Food and Drug Administration’s Center for Food Safety and Applied Nutrition (Supplementary Table 1).

### Phylogenetic Analysis

We assembled 15,580 *S. enterica* genome sequences with SPAdes v3.13.0 (Bankevich et al., 2012) and used SeqSero v1.0.1 (Zhang et al., 2015) to predict their serotypes. We selected 11,701 taxa for further analysis that represent the 29 serotypes with at least 100 isolates (Supplementary Table 2). We defined open reading frames with PROKKA v1.12 (Seemann, 2014) and used BLAST v2.7.1+ (Altschul et al., 1990) to find 1,152 loci that comprise an extended multi-locus sequence typing (MLST) scheme (Pettengill et al., 2016). Sequence data were aligned with MAFFT v7.305b (mafft -adjustdirection infile > outfile; Katoh and Standley, 2013). Parsimony informative single-nucleotide polymorphisms (SNPs) were identified and concatenated into a single alignment with FASconCAT-G v1.04 (FASconCAT-G_v1.04.pl. -o -j -s; Kuck and Longo, 2014). The supermatrix was phylogenetically analyzed with FastTree v2.1.11 SSE3 (FastTreeMP -fastest -nt -gtr < FcC_supermatrix.fas > tree; Price et al., 2009, 2010). The resulting tree was edited with FigTree v1.4.4.^3^

### Evolutionary Rate Measurements

We estimated evolutionary rates for 22 lineages that are comprised of at least 100 taxa. We generated lineage-specific phylogenetic analyses using the CFSAN SNP Pipeline (Davis et al., 2015). We then investigated lineages within those trees with TempEst v1.5.3 (Rambaut et al., 2016) to determine which exhibit clock-like behavior and to remove long-branches. Those lineages with clock-like behavior were analyzed further. We generated alignments of assemblies for each clock-like lineage with SKA v1.0, using default settings, and identified regions of recombination with Gubbins v1.4.5 (-first-tree-builder fasttree -tree-builder fasttree -first-model JC -model GTRCAT; Croucher et al., 2015). Regions of recombination were masked and the resulting alignments were analyzed with BEAST v2.6.2, using default settings (Bouckaert et al., 2019). We used the General time reversible (GTR) nucleotide substitution (Tavaré, 1986) model with both the Strict and Relaxed (Drummond et al., 2006) Log Normal clock models and the Coalescent Constant (Drummond et al., 2005) demographic model for 10^8^ generations, sampling every 5,000 generations. BEAST outputs were visualized with Tracer v1.7.1 (Rambaut et al., 2018). BEAST runs with effective sampling sizes of at least 200 were analyzed further. For four lineages, both the Strict and Relaxed models had effective sampling sizes of at least 200. In these cases, nested sampling was used to select the best-fitting models (Bouckaert et al., 2019). The numbers of SNPs per year were calculated by multiplying the rates estimated with BEAST and the numbers of unmasked sites in the alignments. The slowest rates are reported for those serotypes in which multiple lineages were analyzed.

## Results and Discussion

**Figure 1:**
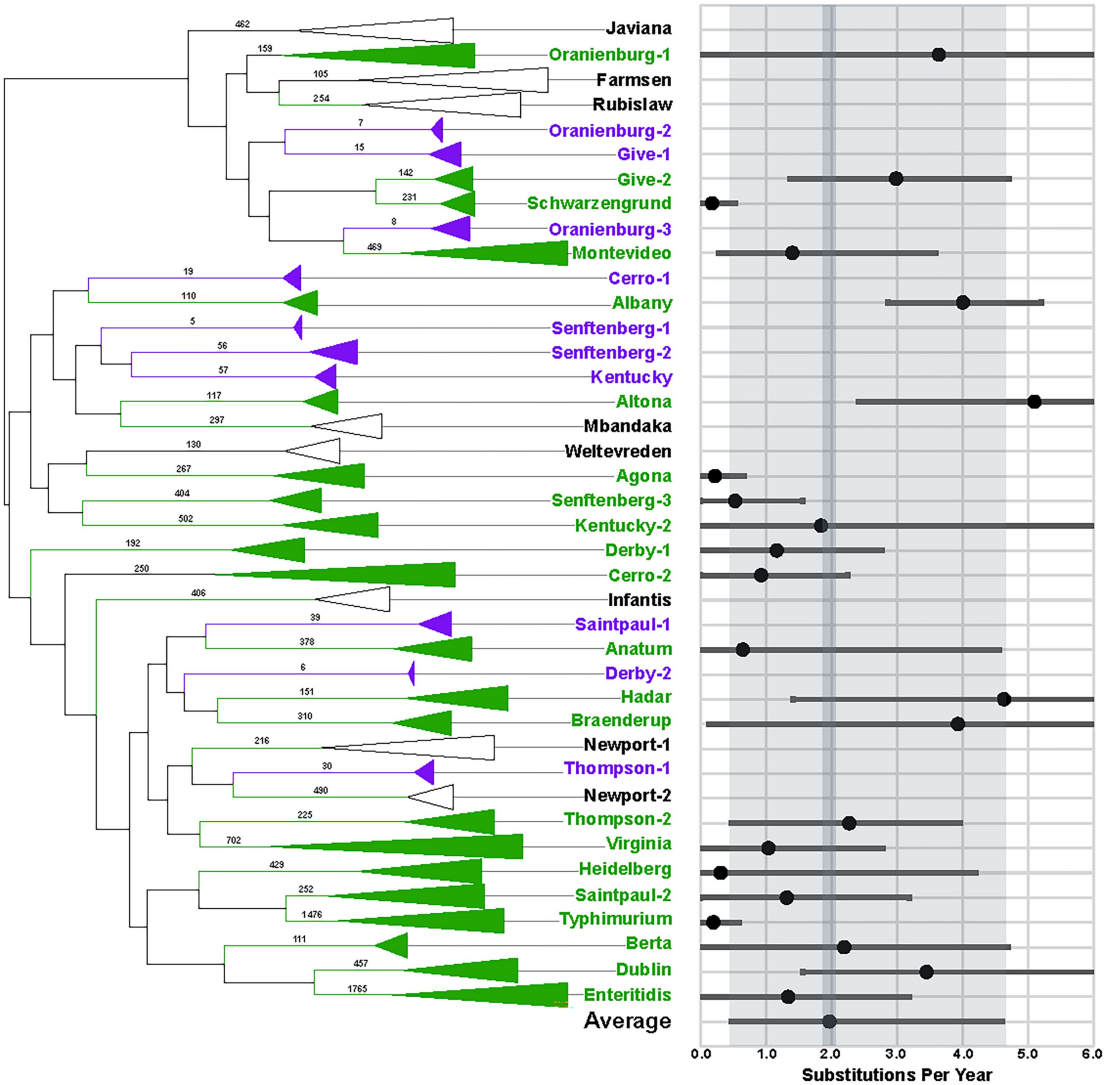
Phylogeny and evolutionary rate measurements of 29 *Salmonella enterica* serotypes. Values on branches of the phylogenetic tree indicate the numbers of taxa that comprise each clade. Green labels indicate that ≥ 100 taxa were available for evolutionary analyses, while purple labels show clades with < 100 taxa that were not analyzed. Clades that have ≥ 100 taxa but did not exhibit clock-like behavior are labeled with black. Results of BEAST analyses are indicated in the graph with black dots. Bars indicate 95% HPD values. Gray shading shows the averages of the serotypes.

We identified the most common *S. enterica* serotypes that were isolated from food and environmental samples in the United States and submitted to the NCBI by the US Food and Drug Administration from 2014 to 2019. We then phylogenetically analyzed the WGS data (Figure 1). Interestingly, we found that 24.2% (7/29) of the serotypes examined are polyphyletic (Cerro, Derby, Give, Oranienberg, Saintpaul, Senftenberg, and Thompson), which has been documented elsewhere (Timme et al., 2013) and further supports that serotypes are not always reliable for estimating genomic similarity (Wattiau et al., 2011).

We estimated evolutionary rates for 22 lineages (Figure 1, green labels and Supplementary Table 3). The average evolutionary rate measured is 1.97 single-nucleotide polymorphisms (SNPs) per year (Figure 1, dark gray shading), with an average highest posterior density (HPD) interval of 0.48-4.61 SNPs/year (Figure 1, light gray shading). The slowest evolutionary rate is 0.18 SNPs/year for *S*. Schwarzengrund (HPD 0-0.53), while the fastest is 5.10 SNPs/year for *S*. Altona (HPD 2.41-7.72). However, since most of the rates measured fall within the average HPD interval, disparities between lineages are less likely to represent true evolutionary differences than to reflect the variability inherent in rate estimates. Thus, we propose that the average of 1.97 SNPs/year be used for determining whether genomes match, while being mindful that evolutionary rates for lineages are likely to vary, depending upon the conditions that they are exposed to.

**Figure 2:**
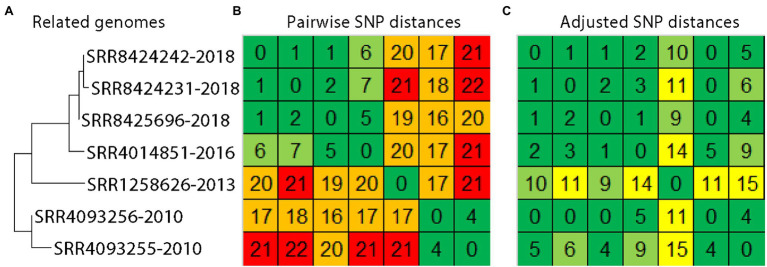
Phylogenetic and pairwise SNP analysis of *Salmonella enterica* collected from the same facility over time. A phylogeny **(A)** and pairwise SNP distance matrix **(B)** was generated with the CFSAN SNP pipeline. Distances were adjusted manually to reflect the time spans separating collections at the rate of 1.97 SNPs/year **(C)**.

These results can be used when establishing matches between *S. enterica* genome sequences. For instance, evolutionary rates can be applied to genome sequence data that were collected at different times. As a test case, we used the evolutionary rates observed here to adjust SNP distances for a real-life genome sequence data-set (Figure 2A). *Salmonella enterica* were isolated from samples that were taken from the environment of a food processing facility and products that originated from that facility. The samples were collected over a time span of 8 years. *Salmonella enterica* that were collected in 2010 are estimated to be 16-22 SNPs distant from *S. enterica* collected from the same firm in 2018 (Figure 2B, orange and red squares). These SNP distances may obscure the true relationships between genomes, being beyond the range that may often be considered when assessing matches. However, adjusting SNP distances by the average evolutionary rate of 1.97 SNPs/year (i.e., initial SNP distance-[1.97 SNPs/year*(collection date difference in years)] = adjusted SNP distance, with a minimum of 0 SNPs) yields a range of 0-6 SNPs (Figure 2C, yellow and green squares), which more accurately reflects their shared origin.

## Conclusion

As genome sequence databases continue to grow, evolutionary analyses are increasingly important for assessing matches between isolates that are separated by ever greater gaps in time. By applying an evolutionary rate of 1.97 SNPs per year, the time spans separating sample collections can be accounted for. The rate proposed here provides general guidance but should not be used in a strict manner, since conditions in individual cases or lineages may vary. This approach will help to elucidate relationships between bacteria, even as changes accumulate, and to reduce bias that may be introduced when comparing WGS data.

## Data Availability Statement

The dataset supporting the conclusions of this article are available in the National Center for Biotechnology Information repository. BEAST XML files are available at figshare (https://doi.org/10.6084/m9.figshare.19617234).

## Author Contributions

AP, HR, and JP conceived the study and wrote the manuscript. AP performed the analyses. HR and JP provided materials and funding. All authors contributed to the article and approved the submitted version.

## Funding

This study was funded by the US Food and Drug Administration, Center for Food Safety and Applied Nutrition.

## Conflict of Interest

The authors declare that the research was conducted in the absence of any commercial or financial relationships that could be construed as a potential conflict of interest.

## Publisher’s Note

All claims expressed in this article are solely those of the authors and do not necessarily represent those of their affiliated organizations, or those of the publisher, the editors and the reviewers. Any product that may be evaluated in this article, or claim that may be made by its manufacturer, is not guaranteed or endorsed by the publisher.

## Footnotes

^1^ncbi.nlm.nih.gov/pathogens/organisms/

^2^ ncbi.nlm.nih.gov/pathogens

^3^ http://tree.bio.ed.ac.uk/software/figtree
